# The role of intracellular protein *O*-glycosylation in cell adhesion and disease^☆^

**DOI:** 10.1016/S1674-8301(11)60031-6

**Published:** 2011-07

**Authors:** Meryem Bektas, David S. Rubenstein

**Affiliations:** a Department of Dermatology,; b Department of Pharmacology,; c Lineberger Comprehensive Cancer Center, University of North Carolina-Chapel Hill School of Medicine, Chapel Hill, North Carolina 27599-7287, USA.

**Keywords:** *O*-glycosylation, *O*-GlcNAc, diabetes, cancer, cell adhesion

## Abstract

Post-translational protein modification, including phosphorylation, is generally quick and reversible, facilitating rapid biologic adjustments to altered cellular physiologic demands. In addition to protein phosphorylation, other post-translational modifications have been identified. Intracellular protein *O*-glycosylation, the addition of the simple sugar *O*-linked N-acetylglucosamine (*O*-GlcNAc) to serine/threonine residues, is a relatively recently identified post-translational modification that has added to the complexity by which protein function is regulated. Two intracellular enzymes, *O*-GlcNAc transferase and *O*-GlcNAcase, catalyze the addition and removal, respectively, of *O*-GlcNAc to serine and threonine side-chain hydroxyl groups. Numerous proteins, including enzymes, transcription factors, receptors and structural proteins have been shown to be modified by intracellular *O*-glycosylation. In this review, the mechanism and relevance of *O*-GlcNAc protein modification are discussed in the context of cell adhesion and several representative diseases.

## INTRODUCTION

Most glycoproteins are made of *N*- or *O*-linked complex sugar residues[1],[2]. Traditionally, glycosylated proteins were thought to be localized extracellularly, including the cell-surface and luminal compartments. Different tissues display unique cell-surface glycosylation patterns that, for example, can be altered during development[1],[3]. In lymphocytes, the cell-surface glycosylation pattern changes according to cell function and activation status[4],[5]. Changes in the cell-surface glycoprotein composition were also observed in malignant transformation leading to a more invasive cell phenotype[6]–[8].

Torres and Hart changed this paradigm in 1984 in a study initially designed to identify correlations between lymphocyte cell surface glycoprotein sugar composition and lymphocyte function, metabolic and developmental stage. In these studies, they made use of highly purified bovine milk galactosyltransferase to add radiolabelled galactose to terminal N-acetylglucosamine residues[9]. As predicted by their initial hypothesis, they noted that thymocyte, B- and T-lymphocyte cell-surface saccharide composition did indeed differ. Unexpectedly, they identified a pool of intracellular proteins containing *O*-glycosidically linked GlcNAc monosaccharides[9]. This had not been described previously. Thus, these experiments led to the discovery that intracellular proteins are modified and regulated by the addition of the monosaccharide *N*-acetylglucosamine[9],[10].

Whereas complex glycosylation of cell surface proteins has been known for a long time, simple protein modification resulting from addition of one sugar residue appearing throughout the cell, particularly nuclear and soluble cell fractions, is relatively new[10],[11]. Hart and colleagues[12] subsequently demonstrated that *O*-GlcNAc modification is dynamic, reversible and short lived and could therefore serve a regulatory function. Moreover, further studies showed that many proteins have several possible *O*-glycosylation sites where the GlcNAc sugar residue is attached via the hydroxyl side chain of serine and threonine. *O*-glycosylation provides an additional means of regulating proteins. Serine and threonine residues can therefore exist in three states: they may be unmodified or either be phosphorylated or *O*-GlcNAcylated. As with phosphorylation, *O*-glycosylation can regulate protein conformation as well as facilitate reversible multimeric protein assembly and stability[13],[14].

In contrast to the numerous kinases and phosphatases that regulate protein phosphorylation, there is a single enzyme responsible for catalyzing protein *O*-glycosylation and a single enzyme for removing the *O*-GlcNAc modification. Shortly after the discovery of *O*-glycosylation, the enzyme responsible for adding *O*-linked *N*-acetylglucosamine to proteins was purified from rat liver extracts and named uridine diphospho-*N*-acetylglucosamine:polypeptide β-*N*-acetylglucosaminyltransferase (*O*-GlcNAc transferase, OGT)[15]. OGT is highly conserved among *C. elegans*, rats and humans sharing 65% amino acid identity. OGT is essential for embryonic stem cell survival; therefore, OGT knockout mice are not viable[16]. OGT is ubiquitously expressed in all tissues with particular enrichment in the brain and pancreas[14]. OGT has several tetratricopeptide repeats (TPRs) at the C-terminus. TPRs are important for protein-protein interactions and substrate recognition. Similar to OGT, *N*-acetyl-β-D-glucosaminidase, the enzyme that removes the *O*-GlcNAc modification (*O*-GlcNAcase, OGA) is localized to the cytoplasmic and nucleoplasmic fractions. The cloned sequence of OGA was found to be identical to the meningioma expressed antigen 5 (*MGEA5*) gene, previously identified in human meningiomas and described as a hyaluronidase[17]. Human OGA shares 55% and 43% homology with that of the fruit fly *Drosophila melanogaster* and the nematode *C. elegans*, respectively. The protein is ubiquitously expressed in human tissues with the highest expression in the brain, placenta and pancreas[18].

Intracellular protein *O*-glycosylation likely functions as a nutrient sensor providing a biochemical switch to enable the cell to adapt its physiology depending upon the presence of low, normal, or high glucose levels. In support of this hypothesis, a variety of key cellular regulators, including transcription factors, enzymes, signaling and structural proteins, are modified by *O*-GlcNAc. Since the discovery of *O*-GlcNAc modification, hundreds of proteins have been described to be regulated by this post-translational modification including several regulatory and structural proteins that have been implicated in human diseases. A few examples are described below.

## PROTEINS REGULATED BY POST-TRANSLATIONAL MODIFICATION

### Transcription factors

***Sp1*** was the first transcription factor described to carry multiple *O*-glycosylation sites. Sp1 is a ubiquitous transcription factor that belongs to the family of Sp/XKLF (specificity protein/Krüppel-like factor) family of zinc-finger motif containing transcription factors[19]. It activates transcription of a wide variety of viral and cellular genes that contain at least one Sp1 binding GC/GT-box in their promoter region[20]. These genes are commonly thought of as housekeeping genes. It was shown that *O*-glycosylated Sp1 is transcriptionally more active than unglycosyated Sp1[21]. Additionally, glycosylation was suggested to stabilize Sp1 against proteasomal degradation[22]. When cells are deprived of glucose, Sp1 is hypoglycosylated and unstable. Sp1 may function as a nutrient sensor and adjust the gene regulation of proteins according to the nutrition status of the cell. Kudlow *et al*.[23] and Zhang *et al.*[24] later demonstrated that the increased instability of hypoglycosylated Sp1 was not due to glycosylation level of Sp1 but to the finding that glycosylation directly inhibits proteasome function.

In conditions of nutrient shortage, gene expression by Sp1 is down-regulated to preserve nutrients[22],[25]. In this context it should be noted that many Sp1 regulated genes are involved in diabetes. High glucose or glucosamine levels drive formation of UDP-N-acetylglucosamine (UDP-GlcNAc), the sugar donor substrate of OGT. Under high glucose conditions, Sp1 becomes hyperglycosylated. *O*-glycosylation can modify protein-protein interaction; therefore, *O*-glycosylation of Sp1 may change its interaction with other proteins of the transcription machinery in such a manner that it favors the expression of diabetes related genes.

In addition to *O*-glycosylation, Sp1 can be phosphorylated at multiple sites, primarily serine and to a lesser extent threonine, but not tyrosine residues. Phosphorylation is regulated by a growing list of kinases and phosphatases that are stimulated by diverse signaling pathways. Increased phosphorylation has been linked to reduced as well as enhanced transcriptional activity, likely dependent on the phosphorylation site and the studied cell-type[26]. Adding to the complexity of Sp1 regulation, Haltiwanger and colleagues[27] demonstrated that some phosphorylation sites on Sp1 might be reciprocally regulated by *O*-glycosylation. Cellular protein *O*-glycosylation levels were increased by selective inhibition of OGA, resulting in increased modification of Sp1 by *O*-GlcNAc with a concomitant decrease in Sp1 phosphorylation[27]. The decrease in phosphorylation, nonetheless, was smaller than the increase in *O*-glycosylation, which led to the conclusion that not all Sp1 phosphorylation sites were inevitably reciprocally regulated by *O*-glycosylation.

***C-myc*** is a nuclear phosphoprotein that regulates gene transcription in cell proliferation, differentiation and cell death. It is also a proto-oncogene that is frequently deregulated in skin and other cancer types[28],[29]. It was one of the first well-characterized targets of *O*-glycosylation. C-myc is *O*-glycosylated at threonine 58, which is also a phosphorylation site[30]. Threonine 58 is frequently mutated in human lymphomas and its modification by phosphorylation or glycosylation depends on external cell stimuli. Serum-starvation of HL-60 cells led to the accumulation of *O*-glycosylated c-myc; in contrast, serum stimulation increased threonine 58 phosphorylation[31].

In keratinocytes, c-myc plays a prominent role in stem cell proliferation and differentiation along the epidermal lineage[32]. One of the known upstream effectors/regulators of c-myc is plakoglobin. Interestingly, plakoglobin is also *O*-glycosylated as described below in more detail. While plakoglobin over-expression in normal keratinocytes suppresses c-myc, in other cell types it leads to strong c-myc expression[33],[34]. The ultimate effect of plakoglobin on c-myc may be determined by its ability to form complexes with a variety of different proteins. Plakoglobin has been shown to recruit multiple kinase and phosphatase receptors[35]. Therefore, posttranslational modifications such as phosphorylation and/or *O*-glycosylation likely regulate the selective complex formation that results in either suppression or stimulation of c-myc by plakoglobin.

### Cell adhesion/structural protein

***Plakoglobin*** functions in both cell-cell adhesion, as a component of both adherens junctions and desmosomes[36], and in cell signaling. In adherens junctions, plakoglobin binds to the cytoplasmic region of cadherin transmembrane cell adhesion proteins, functioning to link cadherin to α-catenin and the actin cytoskeleton. In the desmosome, plakoglobin binds to the cytoplasmic domain of desmoglein transmembrane cell adhesion proteins to form a complex with other proteins, including desmoplakin and plakophilin that similarly link the desmosome adhesive structure to the cytoplasmic keratin intermediate filament cytoskeleton. Plakoglobin also functions in cell signaling as a component of the Wnt signal transduction pathway. Wnt signaling has been extensively studied for its role in development and patterning of tissues and organs. It is a pathway that is highly conserved among different organisms[37]. Importantly, deregulation of Wnt signaling can lead to cancer. Wnt signaling is initiated by Wnt ligand binding to frizzled receptors. This binding triggers a signaling cascade that culminates in the stabilization of non-junctional cytoplasmic β-catenin, which subsequently translocates to the nucleus and activates transcription of genes that contain lymphoid enhancer factor/T-cell factor (LEF/TCF) elements in their promoters. Among the responsive genes are *c*-*myc*, *cyclin*-*D* and *metalloproteinase*, all of which play a role in cancer. In the absence of Wnt, β-catenin is targeted for ubiquitin-dependent degradation[36]. Due to structural similarities, plakoglobin binds to some of the same proteins as β-catenin. Thus, plakoglobin competes with β-catenin for binding partners and can function to down-regulate β-catenin's impact on Wnt/β-catenin-LEF/TCF signaling[36],[38],[39].

In fulfilling its multiple functions, plakoglobin differentially interacts with numerous proteins; this process is regulated in part by selective post-translational modification of plakoglobin. Plakoglobin has a single site for *O*-GlcNAc modification in the *N*-terminal region that lies within the glycogen synthase kinase-3β (GSK-3β) phosphorylation consensus sequence, suggesting that *O*-glycosylation might potentially antagonize GSK-3β mediated plakoglobin phosphorylation[35]. Studies carried out in our laboratory demonstrated that *O*-glycosylation of plakoglobin was indeed associated with decreased plakoglobin phosphorylation and increased plakoglobin protein levels[40]. In these experiments, intracellular protein *O*-glycosylation was increased by driving over-expression of OGT in keratinocytes. OGT over-expression increased plakoglobin protein levels. The effects of OGT on plakoglobin were not occurring via effects on gene transcription nor translation because the protein translation inhibitor cyclohexamide failed to prevent the OGT mediated increase in plakoglobin protein. Using two dimensional gel electrophoresis, we were able to show that driving OGT activity in keratinocytes increased the positively charged plakoglobin isoforms with a concomitant decrease in the negatively charged plakoglobin isoforms. Using phosphatase, we demonstrated that the negatively charged plakoglobin isoforms were phosphorylated since these negatively charged plakoglobin isoforms were markedly reduced in samples treated with phosphatase. These observations indicated that *O*-glycosylation of plakoglobin antagonized plakoglobin phosphorylation. Interestingly, increased levels of plakoglobin in the OGT over-expressing keratinocytes was associated with increased numbers of plakoglobin based adherens junctions and desmosomes, which functionally resulted in a marked increase in keratinocyte cell-cell adhesion[40]. In contrast to the stabilizing effects of plakoglobin *O*-glycosylation on junction formation and cell-cell adhesion, phosphorylation of plakoglobin has been associated with decreased binding to E-cadherin and decreased cell-cell adhesion[41]. Interestingly, disruption of plakoglobin's association with E-cadherin by tyrosine phosphorylation of plakoglobin resulted in translocation of plakoglobin from the cell membrane associated adherens junction to the nucleus. The translocation of tyrosine phosphorylated plakoglobin from the cell membrane to the nucleus suggested that it might modulate gene transcription, a hypothesis supported by additional studies demonstrating that plakoglobin released from the adherens junction competed with β-catenin for association with LEF/TCF proteins and thereby antagonized transcription from LEF/TCF reporter constructs[41]. In the bigger picture, these results suggest that post-translational modification of plakoglobin in part regulates the differing functions of plakoglobin as a component of adherens junctions and desmosomes in its role as a regulator of cell-cell adhesion or as a component of transcriptional regulatory complexes in its role as a signaling protein.

In the epidermis, keratinocytes migrate upward from the basal layer to the spinous and granular layers as they undergo terminal differentiation in the stratum corneum forming a physical barrier of the cornified envelope. For this process of keratinocyte movement/migration and differentiation to proceed in a regulated fashion, fine-tuned regulation of cell-cell adhesion and cell-cell dissociation is critical to allow the cells to selectively dissociate from and re-associate with neighboring cells such that migration through the epidermis can proceed, but without the risk that all the cells of the epidermis lose adhesion to one another. It is likely that selective post-translational modification of plakoglobin facilitates this process. It is interesting to speculate that under conditions of keratinocyte migration, phosphorylation of plakoglobin and decreased cell-cell adhesion are likely to be favored; whereas, *O*-glycosylation of plakoglobin is likely to favor nonmigrating stationary cells and increased cell-cell adhesion.

***Keratins*** are a major protein component of the epithelial cytoskeleton. Keratin isoforms are expressed in a development and differentiation dependent manner and therefore can serve as markers to distinguish epithelial cell lineage. All keratins share a conserved common structure built of a central “rod” domain made of coiled coil α-helix, a non-α-helical “head” and a carboxy-terminal “tail” domain[42]. Post-translational modifications occur on the head or tail domain. Site-specific phosphorylations on keratins, as shown on keratin 8 (K8) and K18, which form obligate heterodimers in simple-type epithelia, regulate keratin association with other proteins, keratin half-life, and keratin filament assembly and organization[43]–[45]. The dynamic reorganization of keratins plays a critical role in a variety of physiologic states including mitosis and in the response to cellular stress[46].

*O*-glycosylation of K8 and K18 was first identified almost two decades ago[47]; however, only recently has it been demonstrated that *O*-glycosylation of K8 and K18 increases their solubility and subsequent degradation[46]. K18 mutants unable to be *O*-glycosylated were more stable than their wild-type counterparts. Addition of *O*-GlcNAc to K8 and K18 increased their ubiquitination, which destined them for subsequent degradation. In many phosphoproteins, phosphorylation and *O*-glycosylation are reciprocal process; however, in K8/K18, both modifications can occur simultaneously, albeit on different residues, of the same protein. The relevance of *O*-glycosylation in normal liver physiology was studied in mice using K18 mutants unable to be glycosylated. Under normal conditions wild-type and mouse carrying the K18 mutant did not show obvious differences; however, upon a stress challenge, the mutant mice were more prone to liver injury than their wild-type counterparts, suggesting a protective role of K18 *O*-glycosylation in hepatocytes[48]. Interestingly, mutations in the K8/K18 genes have been described in both acute and chronic liver disease progression[48],[49].

## CLINICAL RELEVANCE OF *O*-GLYCOSYLATION

Alterations in cell adhesion and its regulation are features of both normal physiologic as well as pathophysiologic processes. During development, levels of cell adhesion junction components are tightly regulated at the gene expression level; however, post-translational modification of junction components also contributes to their proper function. For example, *N*-glycosylation within the extracellular domain of E-cadherin is known to regulate adherens junctions[50]. Low *N*-glycosylation of E-cadherin triggers the recruitment of proteins such as vinculin that stabilize the adherens junctions and further tighten the interaction with the actin cytoskeleton.

Desmosomes are major cell-to-cell adhesion structures of high-mechanical stress tissues such as the skin and heart[51],[52]. The desmosomal structure is a multi-protein assembly that consists of desmosomal cadherins desmoglein and desmocollin as well as plakoglobin, desmoplakin and plakophilin. The adhesive proteins desmoglein and desmocollin undergo intercellular heterophilic as well as homophilic interactions. The desmosome is formed at the plasma membrane and is connected to the intracellular keratin intermediate filament cytoskeleton[53],[54].

Study of the human autoimmune blistering diseases pemphigus vulgaris (PV) and pemhigus foliaceus (PF) has contributed to our understanding of the regulation of desmosome adhesion[55]–[58]. In these diseases, pathogenic autoantibodies bind to the extracellular *N*-terminal domain of the desmosomal cadherins desmoglein 1 and desmoglein 3. The antibodies disrupt desmosome adhesion and lead to loss of keratinocyte cell-cell adhesion and clinical blisters[56],[59],[60]. The exact chronology of how binding of antibodies to desmoglein 1 or 3 results in loss of cell adhesion is still somewhat controversial[59],[61]. What is so far generally acknowledged is that antibody binding triggers a signaling cascade that involves a rapid activation of p38[58]. We demonstrated that p38 activation is an early event crucial to the disease progression by using specific inhibitors that could prevent PV IgG induced blistering in a mouse model of pemphigus[62]. As a consequence of autoantibody binding to desmoglein, desmoglein is internalized into endosomal vesicles that cause it to be depleted from the desmosomes and the keratin intermediate filaments disengage from the desmosomes and retract from the membrane[63]. Importantly, inhibiting p38 blocks both pemphigus IgG induced desmoglein endocytosis and keratin intermediate filament retraction[64],[65].

In addition to their cell-adhesion functions, signaling roles have been identified for several of the desmosome proteins. For example, plakophilin 1 is implicated in regulating translation[66] and plakoglobin is a component of the Wnt signaling cascade[36],[37].

Classical cadherins are known to be involved in tumor initiation and progression. These cadherins can interact with growth factors on the cell surface and thereby regulate them or in turn can be regulated by the growth factors[7]. For example, cadherin isoform switching from *E*- to *N*-cadherin has been described for many different cancer types and often promotes invasion and metastasis. This has been attributed not only to the loss of *E*-cadherin-mediated growth control but also to increased binding of *N*-cadherin to fibroblasts and vascular endothelial cells. *E*-cadherin is known to interact with and inhibit the activity of the epidermal growth factor (EGF) receptor[7],[67],[68]. Furthermore, loss of desmosomal adhesion precedes the epithelial-mesenchymal transition that is strongly implicated in conversion of early stage tumors to invasive cancers[67],[68]. In the context of invasiveness, it has been shown that desmoglein 1, which is predominantly expressed in the suprabasal keratinocytes, inhibits EGFR activity, leading to epidermal differentiation[69]. Therefore, loss or decrease of desmoglein 1 would enhance EGFR activity that would promote cell proliferation and invasion[70].

Many components of the various cell adhesion structures can be phosphorylated and it is feasible that in addition to phosphorylation, some of them may be counter-regulated by *O*-glycosylation. As mentioned above, Hu *et al.*[40] demonstrated that when plakoglobin, an important component of both desmosomes and adherens junctions, is *O*-glycosylated, it increases cell-cell adhesion by enhanced stability of these plakoglobin based junctions. *O*-glycosylation of classical cadherins is not well studied, but Zhu and colleagues[71] studied *E*-cadherin trafficking to and from the membrane under stress conditions. In contrast to the observations of Hu *et al*[40], in which enhanced *O*-glycosylation of plakoglobin stabilized adherens junctions and desmosomes, Zhu and colleagues[71] found that ER stress induced *O*-glycosylation of newly synthesized *E*-cadherin as well as of β-catenin resulted in failure of this newly synthesized *E*-cadherin to be transported to the cell surface with subsequent decreased intercellular adhesion. These observations suggest that the effects of *O*-glycosylation are likely to be variable and context dependent.

### Diabetes mellitus

UDP-*N*-acetylglucosamine (UDP-*N*-GlcNAc), the substrate for OGT mediated protein *O*-glycosylation, is produced via the hexosamine biosynthetic pathway (HBP). Less than 5% of the glucose taken up by the cell enters the HBP[72]. Upon cellular uptake, glucose is phosphorylated to glucose-6-phosphate; a minor fraction of glucose-6 phosphate then enters the HBP as fructose-6-phosphate, which is converted to glucosamine-6-phosphate by the first and rate-limiting enzyme of HBP, glutamine: fructose-6-phosphate-amidotransferase. Glucosamine-6-phosphate is eventually converted to UDP-*N*-GlcNAc[73]. Because of the sensitivity of this pathway to glucose concentrations, changes in glucose concentrations result in changes in the levels of UDP-*N*-GlcNAc. Because a change in HBP is reflective of substrate availability, it has been proposed that this pathway may function as a nutrient sensor[74]–[77], implicating the potential for deregulation of this pathway and/or altered protein *O*-glycosylation in diabetes.

Type 2 diabetes is on the rise in developed nations and is seen, apart from genetic determination, as a disease of over-nutrition and a sedentary lifestyle. A hallmark of type 2 diabetes is insulin resistance, a consequence of increased UDP-GlcNAc levels due to hyperglycemia. Hyperglycemia is associated with increased *O*-glycosylation of a variety of intracellular proteins[78]–[82]. The increased GlcNAc modification of intracellular proteins observed in hyperglycemic states is thought to contribute to the pathology of diabetes. Diabetes in animal models is often induced by the use of the *N*-acetylglucosamine analog streptozotocin, an irreversible inhibitor of OGA, which ultimately causes the death of β-cells of the pancreas[79].

To study insulin resistance, Marshall *et al*.[72] used cultured adipocytes. They demonstrated that glucose going through the HBP may be responsible for the diabetogenic effect of glucose and that sustained high levels of glucose flux through the HBP triggers insulin resistance. Liu and colleagues[82] reached similar conclusions *in vivo* in a study in which pancreatic cell glucosamine levels were elevated by injecting mice with glucose and streptozotocin. Increased glucosamine levels resulted in increased *O*-GlcNAc modification of cellular proteins. Pancreatic β-cells are especially sensitive to changes in intracellular *O*-GlcNAc levels because they express high levels of OGT. Persistent high level of *O*-GlcNAcylation in the pancreas can eventually lead to apoptotic cell death of β-cells[82]. Additional support for an association between protein *O*-glycosylation and diabetes comes from studies in which over-expression of OGT in muscle and adipose tissue caused diabetes in transgenic mouse models[14],[83],[84]. Further support for a role of increased intracellular *O*-glycosylation in diabetes comes from human genetic mapping studies. In a Mexican-American population, a genetic predisposition to adult onset type 2 diabetes was found to be associated with a mutation that causes increased intracellular protein *O*-glycosylation due to an early termination in the gene encoding OGA[85]. As a consequence of the mutation, increased levels of intracellular protein *O*-glycosylation occur due to the failure of the mutant OGA to effectively catalyze the hydrolysis of *O*-GlcNAc from protein substrates.

### Cancer

The transcription factors c-myc (discussed above) and p53 are well-known to play important roles in cancer*. p53* is a key tumor-suppressor gene that is inactivated in a majority of tumors[86],[87]. Under normal conditions, p53 is present at low numbers in the cell; however, upon a stress signal such as DNA damage, p53 rapidly accumulates as a result of post-translational modifications. Phosphorylation of p53 renders its main partner Mdm2 incapable of tightly binding p53, which is necessary to target p53 for degradation *via* the ubiquitin proteasome pathway. In addition to phosphorylation, Yang *et al*.[88] demonstrated that *O*-glycosylation stabilizes p53 protein by preventing phosphorylation of a nearby site required to target p53 for ubiquitin proteasome dependent degradation.

Tumor cells exhibit enhanced glucose uptake and catabolism *via* glycolysis[89]. Beside p53's well-known role in tumorigenesis, it has been shown that p53 is also able to regulate glycolysis[90]. Mutated p53 was shown to increase gene expression of the type II isoform of hexokinase, which phosphorylates glucose to glucose-6-phosphate and is responsible for the high glucose metabolism observed in tumor cells. Nuclear factor-κB (NF-κB) is an important transcription factor in inflammation and is often up-regulated in cancer[91]. Interestingly, loss of p53 increases glycolysis and the activity of NF-κB, which, in turn, is responsible for increased expression of the high affinity glucose transporter GLUT-3[92]. There seems to be a positive loop where glycolysis increases NF-κB activation, which then increases glycolysis. Because of high glucose metabolism, flux of glucose through the HBP is also increased. The increased transcriptional activity of NF-κB was correlated with increased IκB kinase β (IKKβ) activity. IKKβ phosphorylates the inhibitor (IκB) that prevents NF-κB from translocating to the nucleus. The enhanced IKKβ activity, in turn, was achieved by augmented *O*-GlcNAc modification of IKKβ, which prevented the inactivating phosphorylation of a crucial serine residue[93]. It has been speculated that in normal cells p53 may directly bind to IKKβ and contain its activity. Furthermore, p53 increases the protein expression of TIGAR (TP53 induced glycolysis and apoptosis regulator) known to suppress glycolysis[90]. It is, therefore, likely that p53 regulates glycolysis in multiple ways.

In breast cancer tissue and cells, it was shown that *O*-glycosylation is increased by increased OGT protein expression. Interestingly, down-regulation of *O*-GlcNAc/OGT reduced cell growth and invasion. Mice injected with breast cancer cells displayed a reduced tumor growth when the injected cells contained OGT shRNA. The effect of OGT knockdown was attributed to reduced expression of the transcription factor FoxM1, a regulator of genes essential for the G1/S and G2/M cell cycle progression and, therefore, an important player in cancer progression and development[94]. Nevertheless, a direct *O*-glycosylation of FoxM1 as in other transcription factors could not be detected. It is possible that the effect of increased *O*-glycosylation may be indirect through other proteins that regulate the stability of FoxM1.

Adding to the complexity of the biology of this regulatory modification, several groups have now shown that glucose deprivation of cancer cells paradoxically increases *O*-GlcNAcylation of proteins. While in some cases this increase was attributed to elevated protein levels of OGT, in others it was a result of enhanced OGT activity. Interestingly, the increased enzyme activity was accompanied by decreased *O*-GlcNAc modification of OGT itself[95]. In the lung cancer cell line A549, the substrate UDP-N-acetylglucosamine was shown to be replenished by glycogen degradation when an external glucose source was lacking. It is easy to imagine this scenario *in vivo* when the rapidly growing tumor lacks the adequate nutrients to keep up with the high energy demand. As many proteins were shown to be stabilized by *O*-GlcNAc modification, the cell will try to prolong the half-life of intracellular proteins by adding *O*-GlcNAc to prevent their proteasomal degradation, which would otherwise occur under lack of glucose. The authors further show that various types of stress signal such as heat shock, hydrogen peroxide and UVB light, to name a few, increase protein *O*-GlcNAcylation and promote cell survival by increasing the heat shock proteins Hsp70 and Hsp40 expression via elevated OGT expression and activity[77]. The association of heat shock proteins with target proteins may likely limit their further damage by cellular stress. Moreover, in accordance with the effect of *O*-glycosylation in increasing cell tolerance to stress, elevated *O*-glycosylation of the 26S proteasome has been found to inhibit proteasome-mediated protein degradation[24].

In neuroblastoma cells, many stress signals also showed an increase in overall protein *O*-glycosylation[96]. This response was shown to be mediated by AMPK (AMP-activated protein kinase)-induced OGT expression and p38-dependent OGT activation. In these cells, glucose metabolism activates AMPK, which mediates an increase or likely stabilizes OGT mRNA and protein expression. p38, a stress responsive kinase, is activated during glucose deprivation. Activated p38 interacts with OGT and modifies the function of OGT in such a way that specific proteins become better substrates for OGT. A possible explanation could be that activated p38 directly recruits OGT to target sequences or sites[96].

## CONCLUSION

It is becoming increasingly clear that *O*-GlcNAc modification of intracellular proteins regulates a myriad of cellular processes. The ability to modulate this regulatory pathway, either pharmacologically or genetically, has therapeutic implications for pathologies as diverse as cancer and diabetes. Aberrant regulation of protein *O*-glycosylation is likely involved in many human disorders not discussed in this review including Alzheimer's disease[97],[98]. Highly specific inhibitors of OGT and OGA would be valuable tools for further investigation and also serve as potential drugs for human disease. While OGA inhibitors are available, they have off-target effects inhibiting other hexosaminidases. Development of OGA inhibitors has progressed and recently published reports describe a new and highly specific inhibitor of OGA, which was used in the study of Alzheimer disease[99]. In contrast, inhibitors of OGT are currently not available. On a positive note, a recent report describing the crystal structure of OGT in complex with UDP-GlcNAc and in complex with UDP-GlcNAc and a peptide substrate[100], will likely serve to propel forward the search for OGT inhibitors.
